# Next-Generation Multimodality of Nanomedicine Therapy: Size and Structure Dependence of Folic Acid Conjugated Copolymers Actively Target Cancer Cells in Disabling Cell Division and Inducing Apoptosis

**DOI:** 10.3390/cancers11111698

**Published:** 2019-11-01

**Authors:** Manpreet Sambi, Alexandria DeCarlo, Cecile Malardier-Jugroot, Myron R. Szewczuk

**Affiliations:** 1 Department of Biomedical and Molecular Sciences, Queen’s University, Kingston, ON K7L 3N6, Canada; 13ms84@queensu.ca (M.S.); 14ald4@queensu.ca (A.D.); 2 Department of Chemistry and Chemical Engineering, Royal Military College of Canada, Kingston, ON K7K 7B4, Canada

**Keywords:** nanomedicine, FA-DABA-SMA, self-assembly, oncogenic proteins, intracellular disruption, folic receptor alpha, active targeting, drug delivery

## Abstract

Nanomedicine as a multimodality treatment of cancer utilizes the advantages of nanodelivery systems of drugs. They are superior to the clinical administration of different therapeutic agents in several aspects, including simultaneous delivery of drugs to the active site, precise ratio control of the loading drugs and overcoming multidrug resistance. The role of nanopolymer size and structural shape on the internalization process and subsequent intracellular toxicity is limited. Here, the size and shape dependent mechanism of a functionalized copolymer was investigated using folic acid (FA) covalently bonded to the copolymer poly (styrene-*alt*-maleic anhydride) (SMA) on its hydrophilic exterior via a biological linker 2,4-diaminobutyric acid (DABA) to target folic acid receptors (FR) overly expressed on cancer cells actively. We recently reported that unloaded FA-DABA-SMA copolymers significantly reduced cancer cell viability, suggesting a secondary therapeutic mechanism of action of the copolymer carrier post-internalization. Here, we investigated the size and shape dependent secondary mechanism of unloaded 350 kDa and 20 kDa FA-DABA-SMA. The 350 kDa and 20 kDa copolymers actively target folic acid receptors (FR) to initialize internationalization, but only the large size and sheet shaped copolymer disables cell division by intracellular disruptions of essential oncogenic proteins including p53, STAT-3 and c-Myc. Furthermore, the 350 kDa FA-DABA-SMA activates early and late apoptotic events in both PANC-1 and MDA-MB-231 cancer cells. These findings indicate that the large size and structural sheet shape of the 350 kDa FA-DABA-SMA copolymer facilitate multimodal tumor targeting mechanisms together with the ability to internalize hydrophobic chemotherapeutics to disable critical oncogenic proteins controlling cell division and to induce apoptosis. The significance of these novel findings reveals copolymer secondary cellular targets and therapeutic actions that extend beyond the direct delivery of chemotherapeutics. This report offers novel therapeutic insight into the intracellular activity of copolymers critically dependent on the size and structural shape of the nanopolymers.

## 1. Introduction

Targeting strategies of nanopolymers are formulated to enhance the specific distribution of the therapeutic macromolecules in the treatment of cancer. Active targeting mechanisms utilize tumor-specific receptor ligands to achieve a degree of specificity and are therefore utilized as a promising complementary strategy to improve drug delivery. To this end, multifunctional “smart” nanopolymers combine several targeting strategies to increase treatment specificity and reduce systemic toxicities associated with conventional chemotherapeutics.

Recently, we reported on a biocompatible pH responsive, active targeting delivery system, fabricated using folic acid functionalized on an amphiphilic alternating copolymer poly (styrene-*alt*-maleic anhydride) via a biodegradable linker 2,4-diaminobutyric acid (FA-DABA-SMA) [1,2]. Active targeting strategies using folic acid (FA) have gained much attention in cancer because folic acid receptors (FR) are overexpressed on cancerous cells up to two orders in magnitude relative to non-malignant cells [2,3]. Additionally, the self-assembly of the FA-DABA-SMA copolymeric template was designed to be pH responsive, forming amphiphilic nanostructures at pH 7, allowing for the encapsulation of hydrophobic drugs in its interior core. This structure is stable at neutral pH, but collapses under acidic conditions consistent with the tumor microenvironment, thereby releasing the carrier drugs on-site from its core [4]. Nanopolymers can be selectively designed to alter the pharmacokinetic profile and tissue distribution characteristics of drug delivery vehicles. The size, shape and surface modifications, all of which alter the pharmacokinetics and intracellular mechanisms, can be chemically modified such that they can have a significant therapeutic impact in vivo [5].

Investigations into the toxic effects following nanopolymer internalization are minimal as many nanopolymers are designed to be inert delivery vehicles with little or no toxic effects when they release their contents. To this end, Albanese et al. provided a detailed review on the effect of the nanoparticle size, shape and surface chemistry on biological systems upon internalization [6]. Physical attributes of nanopolymers continued to be explored regarding their effects on therapeutic efficacy; however, the consensus remains that the effects and final properties of nanoparticles in the endo-lysosomal vesicles of cells remain unknown. For example, nanoparticles in the intracellular cytosol space can activate several biological functions, including disrupting mitochondrial function, eliciting the production of reactive oxygen species and activation of the oxidative stress mediated signaling cascade [7,8]. Other reports have demonstrated that nanoparticles such as hydrophilic titanium oxide TiO_2_ nanoparticles are oncogenic [9]. It is known that large nanoparticles do not extravasate far beyond the blood vessel, whereas small nanoparticles travel deep into the tumor, but remain there only transiently. Therefore, it is essential to optimize the next generation of nanopolymers focusing on the intracellular therapeutic mechanisms after internalization to successfully translate these drug delivery systems to the clinic.

Recently, we demonstrated for the first time that FA-DABA-SMA can act as a “smart” cancer targeting drug delivery system able to penetrate the inner core of three-dimensional pancreatic and breast cancer tumor spheroids [4]. To elucidate the possibility of toxicity from the polymeric template design, we reported that the empty SMA and functionalized FA-DABA-SMA polymers reduced PANC-1 and MDA MB-231 cancer cell viability using the WST-1 cell proliferation reagent, the data of which indicated a concentration dependent cell death at 48 and 72 hours of incubation [1].

Unexpectedly, these results revealed a previously unknown mechanism(s) of action of the empty FA-DABA-SMA copolymer where it can reduce tumor spheroid volume. We propose that this is achieved through a possible intracellular mechanism of action that targets oncoproteins, thereby inducing apoptosis. The rationale for this is based on a seminal report by Boshnjaku et al. who reported on a novel role for FRα [10]. This report details that ability of FRα to translocate to the nucleus and to act as a transcription factor by binding to the cis-regulatory elements activating transcriptional developmental genes including *HES1* and *FGFR4* [10]. This finding suggests that FRα may influence the regulation of a variety of malignant processes such as cell migration, cell growth and epithelial to mesenchymal transition (EMT) [3]. Given this critical role of the FRα, we propose that FA-DABA-SMA remains bound to FRα and subsequently becomes internalized, leading to disruption of intracellular processes that regulate cell proliferation and survival, ultimately leading to apoptosis. This disruption of the intracellular processes by FA-DABA-SMA binding FRα may, therefore, translate into a potent multimodal therapeutic effect(s) by dysregulating the essential mechanisms of tumorigenesis.

In this report, the FA-DABA-SMA copolymer is found to bind FR, after which it is translocated intracellularly via receptor-mediated endocytosis. Due to the large size and nanostructure of the 350 kDa FA-DABA-SMA copolymer, it can disrupt critical oncogenic processes, including cell proliferation, and induce apoptosis. Here, the internalization of the 350 kDa FA-DABA-SMA was found to reduce cell viability, but also disabled the oncogenic p53, c-Myc and STAT-3 cell survival proteins, inducing apoptosis. The large sized 350 kDa FA-DABA-SMA has a single chain hydrodynamic radius (Rh) of 6 nm and self-assembles into sheets (Rh of the self-assembled SMA nanostructure of 850 nm in water), while the small sized 20 kDa polymer has a single chain Rh of 3 nm and self-assembles into cylinders (Rh of the self-assembled SMA structure of 120 nm in water). The large sized FA-DABA-SMA nanopolymers and not the 20 kDa copolymers were internalized by binding to FR and, subsequently, inhibited intracellular oncogenic proteins. These results support the next-generation multimodality and therapeutic potential of nanopolymers. It is known that nanomedicines conjugated with targeting macromolecules can recognize a specific target, bind and be internalized via a specific mechanism like receptor-mediated endocytosis [11,12]. The novelty of our findings suggests that the critical size and the unique nanostructure of the copolymer enable the active targeting of folic acid receptors to facilitate the internalization, transportation and cellular localization of the delivery vehicle, where it disables oncogenic survival proteins and induces apoptosis. The significance of these findings provides insight into the previously unknown secondary intracellular mechanisms of action of FA-DABA-SMA that may extend beyond simple delivery vehicles previously thought to be inert.

## 2. Results

### 2.1. Folic Acid Receptor Expression on DU-145 Prostate, PANC-1 Pancreatic and MDA-MB-231 Triple-Negative Breast Cancer Cells

**Figure 1:**
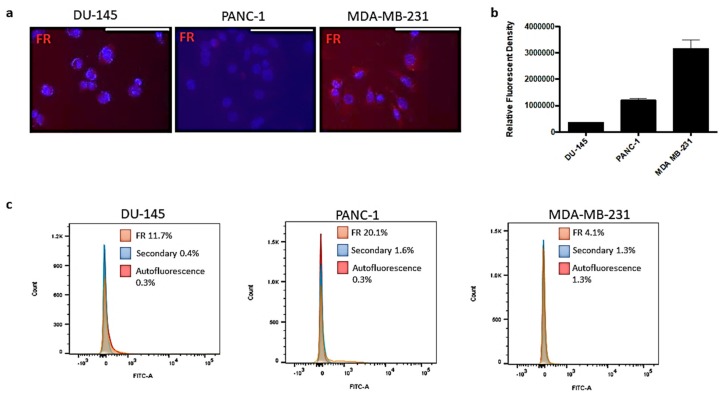
Folic acid receptor (FR) expression levels on DU-145 prostate, MDA-MB-231 breast and PANC1 pancreatic cancer cell lines. (**a**) Immunocytochemistry staining for FR in permeabilized DU-145, MDA-MB-231 and PANC-1 cells. The white scale bar represents 100 µm. Pictures were taken at 400× magnification. Blue DAPI stain represents the nuclei, and red staining is anti-FR antibody followed with AlexaFluor 594 secondary antibody for FR expression. (**b**) Quantification of expression levels by relative density corrected for average background staining of the AlexaFluor 594 secondary antibody. Error bar due to multiple images being quantified (*n* = 3–4). The data are a combination of two independent experiments with similar results. (**c**) Flow cytometry was used to confirm the expression level of the FR. Graphs represent an overlay of FR, secondary only control, and autofluorescence control.

The expression levels of FR were characterized in the prostate (DU-145), pancreatic (PANC-1) and breast (MDA-MB-231) cancer cells. It is well established in the literature that MDA-MB-231 and, to a lesser degree, PANC-1 cells overexpress FR, while DU-145 cells have minimal expression levels [13,14]. In Figure 1a, the immunocytochemistry staining of the FR using the anti-FR antibody showed varying expression levels of the FR on the different cancer cell lines (Figure 1b). 

The MDA-MB-231 cells expressed high levels of FR in comparison to the PANC-1 pancreatic cancer cells. The DU-145 cells expressed minimal levels of FR. These different cell lines allowed for a better understanding of the behaviour of the nanopolymer interacting with the target FR receptor in a range from low to high FR expression levels to better evaluate the efficacy and targeting potential of FA-DABA-SMA. Flow cytometry analyses showed similar trends in FR expression for DU-145 and PANC-1 cells on the cell membrane; however, the expression levels of FR were much lower in the MDA-MB-231 breast cancer cells. The discrepancy between the results may be due to the staining of both external and internalized FR on permeabilized cells (Figure 1a, right) compared to only the surface staining of the receptor (Figure 1c, right). Typically, the majority of FR exists internally and is cycled to the surface dependent on growth related needs. It has been reported that the high expression of FR on MDA-MB-231 breast cancer cell lines may be due to the high metabolic activity of an aggressive invasive ductal adenocarcinoma and, therefore, may require more folate to sustain rapid cell growth [15]. Further research into FR expression has suggested that when cellular growth reaches a plateau and the growth rate slows, an increased expression of FR is observed [16]. Overall, the varying expression levels of the FR in these three cell lines provided valuable information on the efficacy and interactions of the FA conjugated nanopolymers targeting varying expression levels of FR and exerting their therapeutic effects, which will be discussed later.

### 2.2. Effect of Large and Small Sized FA-DABA-SMA Nanopolymers on the Cell Viability of DU-145, PANC-1 and MDA-MB-231 Cancer Cell Lines

**Figure 2:**
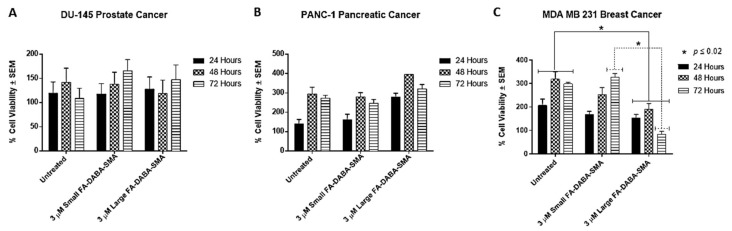
Differential effects of large and small FA-DABA-SMA nanopolymer on cell viability of DU-145 prostate, PANC-1 pancreatic and MDA-MB-231 breast cancer cells. Comparison of the cell viability of (**A**) DU-145, (**B**) PANC-1 and (**C**) MDA MB-231 cell lines at 24, 48 and 72 hours treated with a 20 kDa FA-DABA-SMA (small) and a 350 kDa FA-DABA-SMA (large) nanopolymer using the WST-1 assay. Results were compared by a one-way ANOVA at 95% confidence using Fisher’s LSD test. The data (triplicates) are one of two separate experiments with similar results. The significance is reported in comparison to the untreated control at the respective time point (solid line) and as a comparison between 350 kDa and 20 kDa nanopolymers (dashed line).

Recently, we reported on the therapeutic efficacy of the 350 kDa FA-DABA-SMA and its ability to target FR actively and deliver hydrophobic anti-cancer agents [4,17]. Furthermore, the data in the report revealed that the empty nanopolymers reduced cell viability, penetrated tumor spheroids and decreased their volumes [4]. Here, we questioned whether the size of the nanopolymer could be a contributing factor to these therapeutically important effects of the nanopolymers [1,4,17]. We tested empty large (350 kDa) and small (20 kDa) sized FA-DABA-SMA nanopolymers on DU-145, PANC-1 and MDA-MB-231 cancer cell lines. As shown in Figure 2, both large and small FA-DABA-SMA at the effective dose of 3 μM as determined from our previous studies [1,17] did not affect DU-145 (Figure 2a) and PANC-1 (Figure 2b) cell viability. The MDA-MB-231 cell viability, however, was significantly reduced (*p* < 0.02) by the large nanopolymer when compared with the untreated control cells. These findings confirm the possibility of a novel dual therapeutic mechanism(s) of FA-DABA-SMA. It is proposed that the variation of the expression levels of the FR and the responsiveness of the MDA-MB-231 to the nanopolymer suggest that the binding avidity of the large sheet-like structure of FA-DABA-SMA interaction with FR might have a potent therapeutic effect, leading to FR stimulation and creating an enhanced sensitivity in cancer cells. 

### 2.3. Live Cell Fluorescence Microscopy of Curcumin Loaded 350 kDa FA-DABA-SMA Reveals FR Mediated Endocytosis and Translocation to the Nucleus

**Figure 3:**
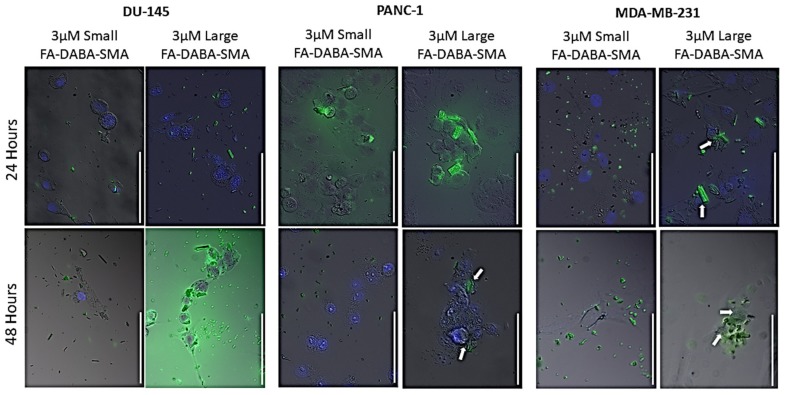
Live-cell fluorescence microscopy of curcumin-loaded FA-DABA-SMA binding FR mediating endocytosis and preferentially targeting the nucleus. Live cell tracking of the large (350 kDa) and small (20 kDa) curcumin loaded FA-DABA-SMA on DU-145, PANC1, and MDA MB-231 cells over 24 and 48 hours. Pictures were taken at 400× magnification. Blue DAPI stain represents the nuclei, and green fluorescence is the curcumin loaded nanopolymers. Results are a combination of two independent experiments with similar results. Arrows indicate nuclei.

To track the nanopolymer’s intracellular activity over 48 hours, the small and large sized FA-DABA-SMA nanopolymers were loaded with curcumin and applied on DU-145, PANC-1 and MDA-MD-231 cancer cells in culture. Within 24 hours, the large FA-DABA-SMA polymer was internalized and appeared to be translocated to the nucleus and released curcumin in the nucleus of MDA MB-231 cells after 48 hours (Figure 3). In contrast, the large nanopolymer was bound to FR on DU-145 prostate cancer cells with no evidence of internalization over 48 hours (Figure 3). As expected, there was no binding and internalization of the small (20 kDa) FA-DABA-SMA nanopolymer in the DU-145 cells. The PANC-1 cancer cells, on the other hand, appeared to interact with the small and large nanopolymer in a manner that was consistent with our previous studies [1,17]. These results suggest that the FR expression levels may not be a significant contributory factor, but more importantly, the size and nanostructure of the copolymer, which subsequently contribute to the therapeutic potential of FA-DABA-SMA, might be a more important factor to consider. To this end, the PANC-1 pancreatic and MDA-MB-231 breast cancer cells were used to investigate further the FR interactions and therapeutic potential of the FA-DABA-SMA copolymer size in delivering hydrophobic chemotherapeutic drugs.

### 2.4. The 350 kDa FA-DABA-SMA Delivers Hydrophobic Chemotherapeutic Drugs More Effectively Than the 20 kDa Copolymer to Reduce Cell Viability

In our initial studies, single chemotherapeutic agents demonstrated a decrease in cell viability; however, recent studies have suggested enhanced effectiveness of a combinatorial approach with nanopolymers [12,18]. Acetylsalicylic acid (ASA), a hydrophobic agent, is known to exert a synergistic effect when administered with conventional chemotherapeutics in several cancers [19,20]. To this end, ASA was chosen to be administered in combination with tamoxifen (Tmx) for MDA-MB-231 breast cancer cells and 5-fluorouracil (5-Fu) for PANC-1 cells.

**Figure 4:**
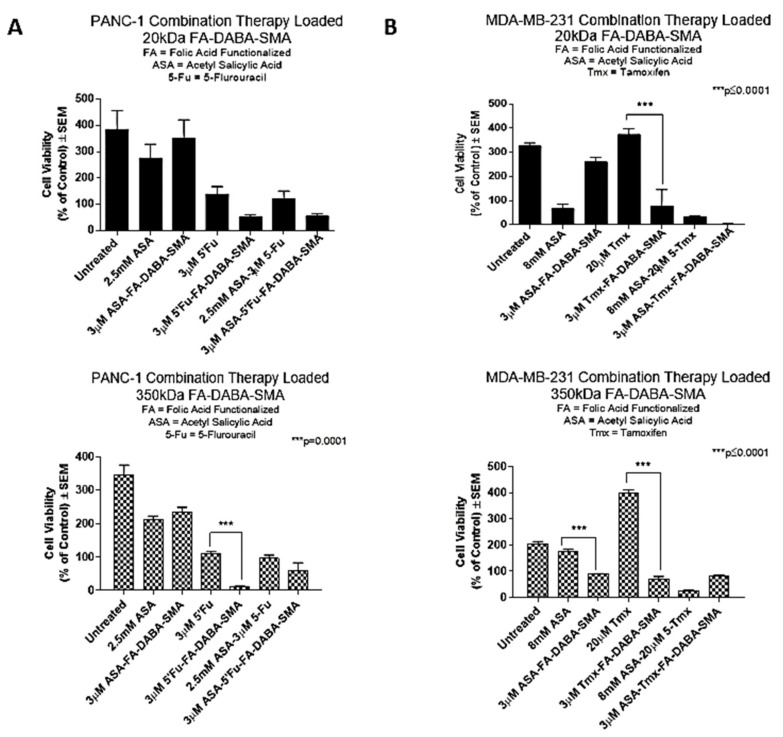
Large nanopolymers deliver hydrophobic therapeutic agents and reduces cell viability more efficiently than small nanopolymers. Comparison of the cell viability of (**A**) PANC-1 and (**B**) MDA-MB-231 cell lines at 72 hours treated with 20 kDa (small) and 350 kDa (large) FA-DABA-SMA nanopolymer loaded with hydrophobic agents, acetylsalicylic acid (ASA), 5-fluorouracil (5-Fu), and tamoxifen (Tmx) given individually or in combination using the WST-1 cell proliferation assay. Results were compared by a one-way ANOVA at 95% confidence using Fisher’s LSD test (*n* = 6). The data are a combination of two independent experiments with similar results. The significance is in comparison to the nanopolymer delivered drug(s) and drug alone at the respective time point.

PANC-1 pancreatic cancer cells were treated with small and large nanopolymers loaded with ASA and 5-Fu or with the respective drugs alone, and the cell viability was measured after 72 hours of treatment. In Figure 4, PANC-1 cells exhibited a significant (*p* < 0.001) decrease in cell viability when treated with the large nanopolymers loaded with 5-Fu compared with the 5-Fu drug alone. However, all other treatment conditions, including a combination treatment, did not show significant differences between free drugs or drug loaded nanopolymers. Drug loaded small nanopolymers also demonstrated no significant results when compared to cells treated with the drugs alone. Compared to the untreated control cells, all treatment groups except for 3 μM ASA loaded small FA-DABA-SMA and 2.5 mM ASA showed significant (*p* < 0.01 to *p* ≤ 0.0001) decreases in cell viability. 

In contrast, MDA-MB-231 cells treated with either ASA or tamoxifen (Tmx) loaded large nanopolymers showed a significant decrease (*p* ≤ 0.0001) in cell viability when compared with the drugs alone. Consistent with previous results, MDA-MB-231 cells treated with Tmx loaded small nanopolymers exhibited a significant decrease in cell viability (*p* ≤ 0.0001) when compared with Tmx alone. Compared to the untreated control cells, all treatment groups except for 3 μM ASA loaded small FA-DABA-SMA and 20 μM Tmx demonstrated significant (*p* < 0.01 to *p* ≤ 0.0001) decreases in cell viability. 

Collectively, these results indicate that the potency of both large and small nanopolymers, when loaded with hydrophobic drugs, may vary depending on the cancer type. For example, the MDA-MB-231 breast cancer cells demonstrated higher sensitivity to the treatment with both small and large nanopolymers when compared with PANC-1 pancreatic cancer cells. Interestingly, the potency of the combination drug therapy when delivered via the nanopolymers was comparable to the drugs alone across both cell lines. For example, tamoxifen is used to treat estrogen receptor positive breast cancer cases, as an important adjuvant hormonal therapy. However, tamoxifen induced side effects have been noted, in particular fatty liver, which is one of the most common side effects among them. Actively targeting cancer cells with drug loaded nanopolymers would upend any of these adverse side effects of the chemotherapeutics. Furthermore, the encapsulation of the hydrophobic drugs in an amphiphilic delivery system is expected to improve the solubility of the drug for enhanced access to cancerous cells. From these results, the size of the nanopolymer does not appear to affect the drug delivery ability of the nanopolymer as both the small and large therapy loaded nanopolymers were significantly able to reduce cell viability in both cell lines. Given these and earlier results, we further investigated the possibility of a novel dual therapeutic role of the large nanopolymers on both PANC-1 and MDA-MB-231 cancer cells. 

### 2.5. Folic Acid-Functionalized Copolymer Disables Intracellular Expression of STAT3, p53 and c-Myc in MDA-MB-231 Breast Cancer Cells 

To determine the possibility of a novel intrinsic therapeutic property of the large sized FA-DABA-SMA copolymer, it was important to consider its effects on critical oncogenic proliferation proteins that promote tumorigenesis.

**Figure 5:**
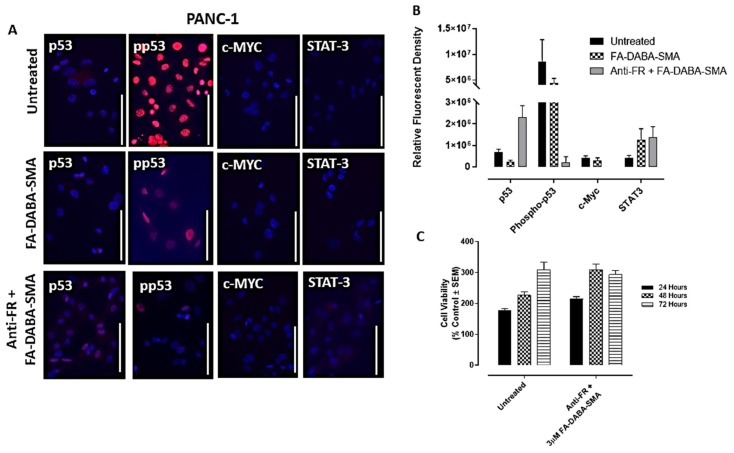
Dual role of FA-DABA-SMA copolymer targeting folic acid receptors (FR) and intracellular Inhibition of STAT3 and p53 and c-Myc in PANC-1 pancreatic cancer cells. (**A**) Immunocytochemistry staining of p53, phospho-p53, c-Myc and STAT3 on the PANC-1 cell line before (untreated), treated with large 350 kDa FA-DABA-SMA copolymer or pretreatment with the anti-FR antibody for two hours followed with large 350 kDa FA-DABA-SMA nanopolymer. The white scale bar represents 100 μm taken at 400× magnification. Blue DAPI stain represents the nuclei, and red staining is a primary antibody for the respective protein expression followed by AlexaFluor 594 secondary antibody. (**B**) Quantification of expression levels by relative density corrected for average background staining of the secondary antibody, mean ± S.E.M. of 3–4 different staining images. Results were compared by a one-way ANOVA at 95% confidence using Fisher’s LSD test for a combination of two independent experiments. (**C**) Comparison of the cell viability of PANC-1 cells’ pre-treatment with the anti-FR antibody for two hours followed by large 350 kDa FA-DABA-SMA nanopolymer at 24, 48 and 72 hours using the WST-1 assay. Results were compared by a one-way ANOVA at 95% confidence using Fisher’s LSD test. The data presented are a combination of two independent experiments with triplicates (*n* = 6).

Here, we treated PANC-1 and MDA-MB-231 cancer cells for 48 hours with large empty FA-DABA-SMA and investigated the effects of this nanopolymer on oncogenic proteins contributing to cell proliferation such as p53, activated phospho-p53, c-Myc and STAT3. In Figure 5a,b, the PANC-1 cells treated with the large empty nanopolymers exhibited a modest and insignificant decrease in expression levels of phospho-p53 along with p53, STAT-3 and c-Myc, when compared with untreated cells.

**Figure 6:**
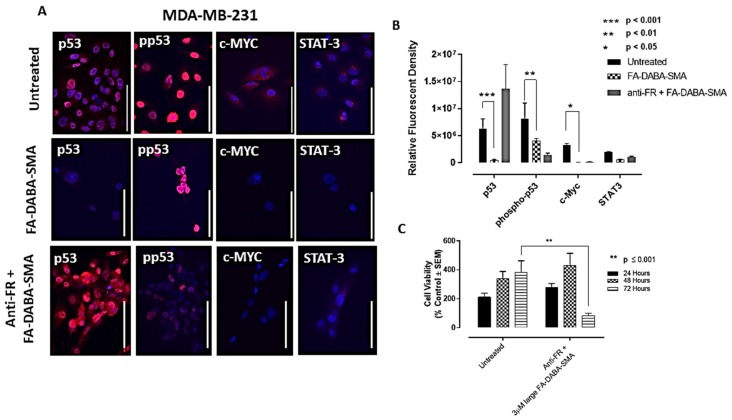
Dual role of FA-DABA-SMA copolymer targeting folic acid receptors (FR) and intracellular Inhibition of STAT3, p53 and c-Myc in MDA-MB-231 breast cancer cells. (**A**) Immunocytochemistry staining of p53, phosphor-53, c-Myc and STAT3 on the MDA-MB-231 cell line before (untreated), treated with large 350 kDa FA-DABA-SMA copolymer or pretreatment with an anti-FR antibody for two hours followed by large 350 kDa FA-DABA-SMA nanopolymer. The white scale bar represents 100 μm taken at 400× magnification. Blue DAPI stain represents the nuclei, and red staining is a primary antibody for the respective protein expression followed by AlexaFluor 594 secondary antibody. (**B**) Quantification of expression levels by relative density corrected for average background staining of the secondary antibody, mean ± S.E.M. of three to four different staining images. Results were compared by a one-way ANOVA at 95% confidence using Fisher’s LSD test for a combination of two independent experiments. (**C**) Comparison of the cell viability of MDA-MB-231 cells’ pretreatment with an anti-FR antibody for two hours followed by large 350 kDa FA-DABA-SMA nanopolymer at 24, 48 and 72 hours using the WST-1 assay. Results were compared by a one-way ANOVA at 95% confidence using Fisher’s LSD test. The data presented are a combination of two independent experiments with triplicates (*n* = 6).

However, consistent with our previous results, the MDA-MB-231 breast cancer cells demonstrated increased responsiveness to treatment with the large empty nanopolymers. These results revealed that all oncogenic proliferation protein levels decreased after treatment of the large (350 kDa) FA-DABA-SMA with a significant decrease (*p* < 0.05) in p53, phospho-p53 and c-Myc expression levels (Figure 6a,b). These results suggest that upon internalization, the FA-DABA-SMA can disrupt the intracellular proliferation activities of critical oncogenic proliferation proteins in breast cancer cells. 

### 2.6. Anti-Folic Acid Receptor Blocking Antibody Reversed the Inhibitory Effects of the FA-DABA-SMA, Leading to an Increase in Expression Levels of p53 and STAT-3

To confirm that the resulting decrease in the expression levels of p53, phospho-p53 and c-Myc was facilitated by the nanopolymer’s intracellular specific inhibitory actions FR, we treated PANC-1 and MDA-MB-231 cancer cells with anti-FR antibody and assessed the effects on cell viability and expression levels of p53, phospho-p53, STAT-3 and c-Myc. As shown in Figure 5c, the PANC-1 cells showed no significant decrease in cell viability with the large empty nanopolymers following anti-FR antibody blocking.

In contrast, the MDA-MB-231 cells, as depicted in Figure 6c, maintained cell growth up to 48 hours, followed by a significant (*p* ≤ 0.0001) decrease in cell viability. This directly contrasts the results presented in Figure 2c, where significant decreases in cell viability following treatment with the large nanopolymers began immediately at the 24 hour time point. This finding may be attributed to the anti-FR blocking antibody being maintained in the presence of the large sized nanopolymer, resulting in a decrease in cell viability. To confirm these findings, immunocytochemistry analyses were performed following 48 hours of treatment with the FR antibody. 

In Figure 5a and Figure 6a (third row), the data revealed the increased expression of p53 in both PANC-1 and MDA-MB-231 cells, respectively, and STAT-3 expression in PANC-1 cells. This contrasts with the pre-treatment data presented in Figure 5a (first and second row), where p53 and STAT-3 expressions in PANC-1 cells were negligible. Similarly, in Figure 6a, the data showed low expression of p53 in MDA-MB-231 cells, whereas with the anti-FR blocking antibody, high expression levels of p53 were observed, as depicted in Figure 6a. Furthermore, the phospho-p53 expression was lower after anti-FR antibody blockage, which contrasts with the high expression levels observed in the data presented in Figure 5a and Figure 6a for PANC-1 and MDA-MB-231 cells, respectively.

Collectively, these results indicate that the activity of the nanopolymer was due to specific inhibitory intracellular interactions, rather than the result of extracellular actions when the FR was inhibited. These findings support the concept of a new therapeutic action of the large FA-DABA-SMA bound to FR in addition to its cargo loaded with hydrophobic chemotherapeutic drugs. 

### 2.7. FA-DABA-SMA Activates Early and Late Apoptosis in PANC-1 and MDA-MB-231 Cells

**Figure 7:**
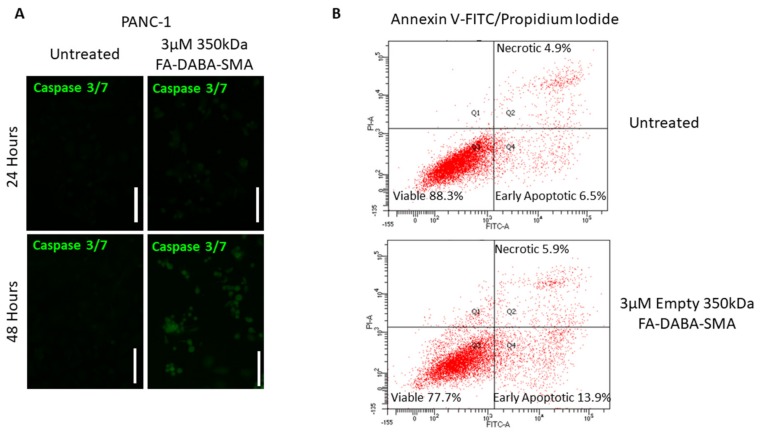
Caspase 3/7 and Annexin-V apoptotic activity post-treatment with 350 kDa FA-DABA-SMA on PANC-1 pancreatic cancer cells. (**A**) Immunocytochemistry staining for caspase on the PANC-1 cell line before after 24 and 48 hours of 3 μM FA-DABA-SMA (350 kDa) treatment. The white scale bar represents 100 μm, and pictures were taken at 400× magnification. Blue DAPI stain represents the nuclei, and green staining is caspase expression. (**B**) Flow cytometry of the Annexin-V apoptosis assay of untreated PANC-1 cells or 48 hours after treatment with 3 μM FA-DABA-SMA (350 kDa). An estimate of ~250,000 cells was collected and stained with Annexin V-FITC/propidium iodide to assess the stage of apoptosis.

To assess the resultant effects of the intracellular inhibition of proliferative oncoproteins, we investigated apoptotic activity following treatment with 3 μM empty 350 kDa nanopolymers in PANC-1 and MDA-MB-231 cancer cells. As shown in Figure 7a, caspase 3/7 activity significantly increased after 48 hours of treatment when compared to the untreated control. Following 48 hours of treatment with nanopolymer, PANC-1 cells entered early apoptosis (13.9%) and late apoptosis/necrotic (5.9%) stages when compared to the untreated cells (early apoptosis 6.5% and late apoptosis/necrotic 4.9%)., as shown in Figure 7b. Furthermore, the number of viable cells in the untreated control was 88.3%, while the number of viable cells in the treatment group was 77.7%. 

**Figure 8:**
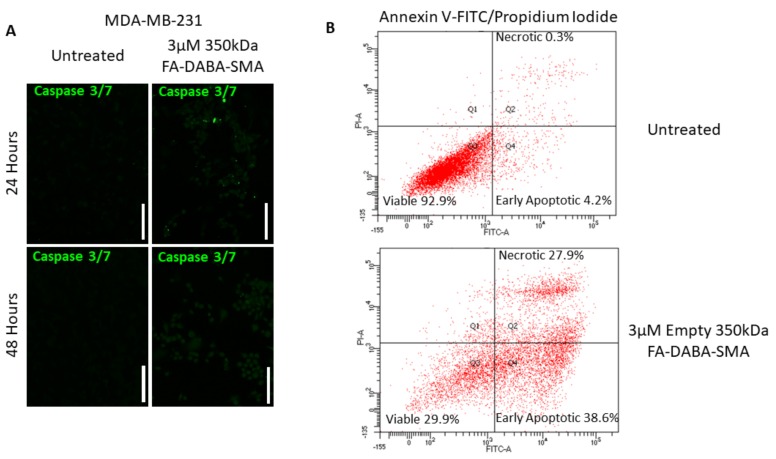
Caspase 3/7 and Annexin-V apoptotic activity post-treatment with 350 kDa FA-DABA-SMA on MDA-MB-231 breast cancer cells. (**A**) Immunocytochemistry staining for caspase on the MDA MB-231 cell line before and after 24 and 48 hours of FA-DABA-SMA (350 kDa) treatment. The white scale bar represents 100 μm, and pictures were taken at 400× magnification. Green staining is caspase expression. (**B**) Flow cytometry of Annexin-V apoptosis assay of untreated MDA-MB-231 cells or 48 hours after treatment with 3 μM FA-DABA-SMA (350 kDa). An estimate of ~250,000 cells was collected and stained with Annexin V-FITC/propidium iodide to assess the stage of apoptosis.

Caspase 3/7 activity did not significantly increase after 48 hours of treatment with 3 µM FA-DABA-SMA when compared to the untreated control in MDA-MB-231 breast cancer cells (Figure 8a). However, upon further investigation, a significant portion of MDA-MB-231 breast cancer cells entered early apoptosis (38.6%) and late apoptosis/necrotic (27.9%) stages when compared to the untreated cells (early apoptosis 4.2% and late apoptosis/necrotic 0.3%), as shown in Figure 8b.

Furthermore, the number of viable cells in the untreated control was 92.9%, while the number of viable cells in the treatment group was significantly reduced to 29.9%. These results indicate the intracellular interactions of FA-DABA-SMA with cell proliferative proteins, and possibly other critical molecules required to maintain cancer cell growth are irreversibly disrupted, leading to apoptosis. The consequences of intracellular protein disruptions were far more pronounced in the MDA-MB-231 breast cancer cells when compared to the PANC-1 cells, suggesting that this novel mechanism of action could represent a novel aspect of intrinsic therapeutic abilities that include cytotoxic activities of internalized nanopolymers that may be cell specific. However, the therapeutic delivery capabilities of the FA-DABA-SMA were relatively consistent between the PANC-1 and MDA-MB-231 cancer cell lines. 

## 3. Discussion

Active targeting of overly expressed receptors and proteins on cancer cells is an important area of cancer research to increase the effectiveness of treatment regimens while at the same time reducing adverse side effects. This approach is equally valid of nanotechnology based drug carriers, which exploit overexpressed receptors to target cancer cells specifically and deliver the therapeutic drugs. Typically, these delivery nanoparticles are designed to be chemically inert and indirectly exert a therapeutic effect by delivering anti-cancer drugs. Advancements in research on the design and unique fabrication of nanopolymers with theranostic properties [21] focus on the physicochemical properties of the polymers, but cannot target cancer specific biological behavior in and around tumors and malignant cells [22]. Although there is robust research progress in the development of nanotechnology, knowledge of their intracellular tracking and endocytic pathway remains limited [5]. Therefore, the understanding of the intracellular mechanisms of nanopolymers is essential when developing the next generation of nano-delivery systems that can be successfully translated in the clinic. As such, this study was designed to further investigate the previously unknown intracellular mechanism of action of a pH responsive FA functionalized amphiphilic alternating copolymer (FA-DABA-SMA) on prostate DU-145, pancreatic PANC-1 and triple-negative breast MDA-MB-231 cancer cells [1]. FA-DABA-SMA has previously been shown to be a successful drug delivery system by actively targeting and penetrating tumor spheroids [4]. However, this study revealed a previously unknown therapeutic mechanism of action of empty FA-DABA-SMA in disabling cancer cell proliferation and survival.

The chemical design of the FA-DABA-SMA nanopolymer was initially fabricated to be pH-responsive, capable of self-assembly into ordered sheet like structures (large 350 kDa) or cylindrical structures (20 kDa) in a size dependent manner. Although this nanopolymer was designed to be inert when it was not loaded with chemotherapeutics, this study confirmed that the size and structure of FA-DABA-SMA contributed to its unusual intracellular therapeutic activity when unloaded. The influence of size and shape needs to be further investigated because the structure of the nanopolymer may be critical in facilitating the binding activity to the FR in order to promote the intrinsic therapeutic potential of FA-DABA-SMA. 

**Figure 9:**
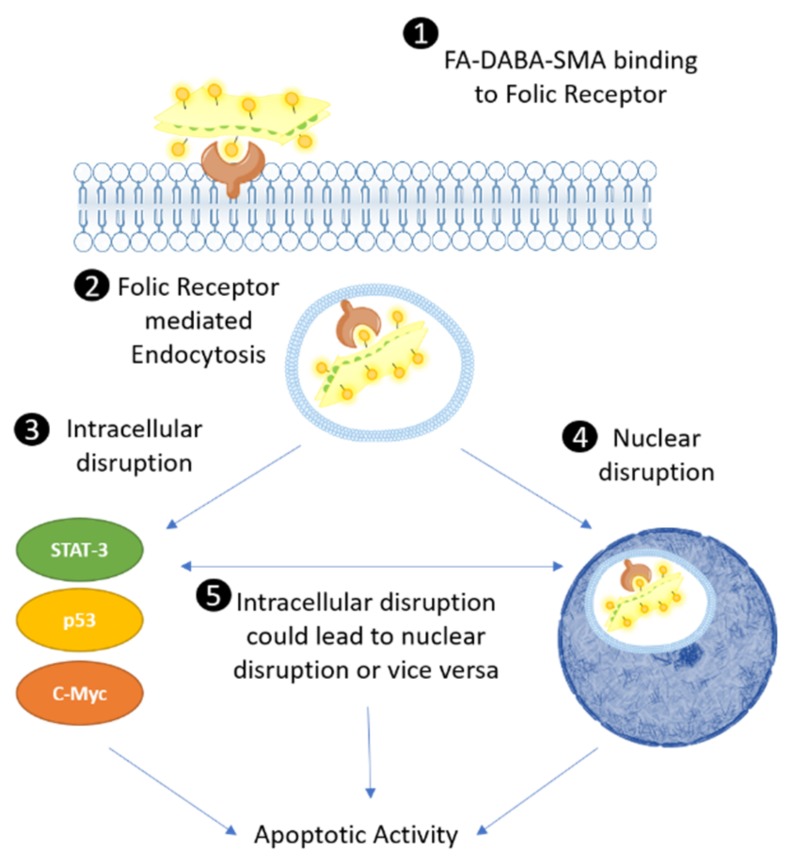
Proposed novel intrinsic therapeutic mechanism of FA-DABA-SMA. (1) FA-DABA-SMA binds to FRα. (2) Through receptor mediated endocytosis, the FRα-FA-SMA-DABA complex is internalized, where it could have two possible intrinsic therapeutic effects. It either (3) remains in the cytoplasm and disrupts the activity of key proliferation proteins such as STAT-3, p53 and c-Myc (and other proteins that maintain cancer growth and proliferation) or (4) the complex is translocated to the nucleus given the role of FRα as a transcription factor. Since FA-DABA-SMA is relatively large (350 kDa), the nanopolymer could theoretically block the binding of additional transcription factors, thus disrupting transcriptional processes of essential oncogenic proliferation proteins, resulting in the reduction of cell viability and activating apoptotic activity. Alternatively, (5) intracellular disruptions of critical cell proliferation proteins could lead to apoptotic events to be activated in the nucleus or, conversely, nuclear disruptions could lead to disruptions in the activity of critical oncogenic proteins. Overall, Steps 3, 4 and 5 could individually or collectively culminate and lead to the activation of apoptotic activity.

Understanding the structure of FRα and its interactions with FA is important to consider in providing a potential explanation for the inherent therapeutic activity of FA conjugated nanopolymers. Under normal conditions, FA binds to FRα and is internalized through receptor mediated endocytosis [23]. Following internalization, the acidic environment of the endosome results in the release of folate from the receptor, followed by transportation to the cytoplasm by a proton coupled folate transporter [24]. Concerning the molecular interaction between FA and FRα, FA docks in a deep binding pocket of the receptor in a perpendicular orientation [24]. The critical finding by Chen et al. was that the pterin group located FRα binding pocket is critical in anchoring the folate to the receptor located in the binding pocket, while a glutamate group sticks out and can be readily conjugated with drugs. Given the critical role of FA orientation when binding to FRα, it is not surprising that the size and structure of FA conjugated nanopolymers is an important parameter to consider in order to facilitate adequate binding and subsequent internalization. However, the mechanistic features and properties for many nanopolymer delivery systems occurring after internalization remain unknown. These mechanistic profiles of nanopolymers may rely on the specific target that influences the pathophysiology of the tumour, including the accumulation, distribution, retention and efficacy of nanopolymers [22]. It is essential that key characteristics of nanopolymers, including size, shape and surface profiles be engineered to improve biodistribution and clearance of the nanopolymer for specific cancers and their characteristic tumor biology. For the first time, our findings support a next-generation multimodal potency of action of a nanopolymer, which depends on the cancer cell types. The MDA-MB-231 breast cancer cells were found to be most sensitive to the secondary multimodal effects, as depicted in Figure 9.

The proposed intracellular mechanism of FA-DABA-SMA bound to FR exhibited two possible therapeutic effects (Figure 9). It either remained in the cytoplasm and disrupted the activity of STAT-3, p53 and c-Myc and possibly other intracellular proteins, followed by translocating to the nucleus and inhibiting the FRα transcription factor from binding to the cis-regulatory elements and activating the transcription of developmental genes including *HES1* and *FGFR4*. This nanopolymer may affect multiple processes involved in tumorigenesis through varying, but similar mechanisms, such as disrupting epithelial to mesenchymal transition (EMT) via *HES1* inhibition [25]. HES1 is positively correlated with the levels of stem cell markers, suggesting that it may play a critical role in tumorigenesis through increasing the number of stem cell like cancer cells (CSCs) associated with mesenchymal phenotypes [3]. The large sized FA-DABA-SMA (350 kDa) may also block the binding of additional transcription factors in disrupting the transcriptional processes of essential oncogenic proteins. These resulting cytotoxic effects of the large sized and sheet like structured nanopolymer adds to the next-generation multimodality and therapeutic effects in reducing cancer cell viability and inducing apoptotic cell death.

FA-DABA-SMA is a smart drug delivery system specifically targeting the commonly overly expressed FR on cancer cells. In the present study, the characterization of the FR expression levels on all three cancer cell lines was essential to understand the underlying therapeutic mechanism(s) of the nanopolymer. As indicated in Figure 1a, MDA-MB-231 cells overly expressed FR when compared with DU-145 and PANC-1 cancer cells. The indicated quantification of the relative fluorescent density of the FR expression showed a consistent trend (Figure 1b). To further confirm the expression levels of FR on the cell membrane, flow cytometry analyses were conducted on all three cell lines. Surprisingly, these results were inconsistent with immunocytochemistry staining. The MDA-MB-231 cells are highly metabolically active and reflect an aggressive invasive ductal adenocarcinoma and, therefore, may require more folate to sustain rapid cell growth [15]. A possible explanation for the discrepancy can be attributed to the activity of FR in rapidly growing cells such as MDA-MB-231 [16]. Furthermore, the decreased need for folate when it is present in the environment also affects the FR expression levels. FR exists both on the cell surface and intracellularly dependent on cell specific growth needs, where 50 to 75% of FRα exists in endosomal compartments. However, the events leading to the trafficking of these receptors to the cell membrane remain unknown, and alterations in location could be attributed to growth specific needs or changes in the endocytic pathway events of GPI-anchored proteins [26]. The expression and binding activity of FR are regulated by the location of the receptor and the local environment, including pH, folate concentration and rate of receptor saturation and cycling [27]. The varying expression levels seen in the MDA-MB-231 cells may be a resulting differential receptor cycling, as the nanopolymer consistently targeted these cells. For the MDA-MB-231 breast cancer cells, the decreased membrane expression levels of FR observed in the present data may be due to more rapid cell growth and cell cycling when compared to the higher FR levels on DU-145 and PANC-1 cells. However, this merits further investigation and a deeper understanding of the stimulatory effects of folate and how this may influence the expression of the FR and in turn responsiveness to the action of FA-DABA-SMA. 

Overall, the novel findings of this report highlight the importance of size and structure in possibly conferring intrinsic therapeutic abilities in FA-DABA-SMA that could potentially be extrapolated to other drug delivery vehicles and merit further investigation. Although both the 350 kDa and 20 kDa FA-DABA-SMA copolymers appeared to unload their hydrophobic payloads, only the 350 kDa nanopolymer can disrupt critical oncogenic proteins that are necessary for cancer growth. Furthermore, the activation of early apoptotic events, as well as caspase 3/7 enzymes, suggests that the dual therapeutic activity of the drug-loaded nanopolymers is irreversible and cytotoxic to rapidly proliferating cancer cells. Additional oncogenic proteins and critical survival activities that are necessary for cancer growth such as EMT and the selection of a CSC phenotype also merit further investigation. 

## 4. Conclusions

This report presents next-generation multimodality and therapeutic effects of the size and structure dependence of functionalized folic acid conjugated amphiphilic alternating copolymer actively targeting cancer cells and disabling critical oncogenic proteins controlling cell division and apoptosis. Furthermore, this smart nanopolymer can effectively deliver hydrophobic chemotherapeutics to cancer cells preferentially to decrease the adverse side effects associated with cancer drug treatments. The large size and sheet like structure of FA-DABA-SMA allowed the nanopolymer to possess a previously unknown dual mechanism of disrupting the activity of intracellular oncogenic proteins and inducing apoptosis. The data suggest that FA-DABA-SMA is a potent delivery system capable of accurately targeting highly metabolic cancer cells and overcoming the barriers of conventional chemotherapy approaches. The size and structure of FA-DABA-SMA require further understanding of the full mechanistic potential of FA-DABA-SMA, specifically interacting with FR overly expressed in different cancer types.

## 5. Materials and Methods

### 5.1. Cell Lines and Culture Procedures

DU-145 (ATCC^®^ HTB-81™) is a human prostate cancer cell line derived from a metastatic brain lesion from a 69-year older man with prostate cancer. PANC-1 (ATCC^®^ CRL-1469™) is a human pancreatic cancer cell line whose site of origin was the duct in a 56-year old male with pancreatic ductal adenocarcinoma. PANC-1 has a genetic profile that has been characterized to express *KRAS*, *TP53*, and *CDKN2A* [28]. MDA-MB-231 (ATCC^®^ HTB-26™) is a human triple-negative breast cancer cell line whose site of origin was a metastatic pleural effusion site of a 51-year-old woman with metastatic breast cancer. MDA-MB-231 is a highly aggressive invasive ductal adenocarcinoma and has a genetic profile that expresses *BRAF*, *CDKN2A*, *KRAS*, *NF2*, *TP53*, and *PDGFRA* mutations [29].

All three cell lines were grown in standard culture conditions containing 1× DMEM conditioned medium supplemented with 10% fetal bovine serum (FBS; HyClone, Logan, UT, USA) and 5 μg/mL plasmocin™ (InvivoGen, San Diego, CA, USA). All cells were incubated at 37 °C in a 5% CO_2_ incubator. Cells were sub-cultured as needed (approximately every 4–5 days) using TrypLE Express (Gibco, Rockville, MD, USA).

### 5.2. Reagents

Acetylsalicylic acid (>99% pure, Sigma-Aldrich, Steinheim, Germany) was dissolved in dimethyl sulfoxide (DMSO) to prepare a 550 mM stock solution, which was stored in aliquots at −20°C. Tamoxifen citrate salt (≥99% pure, Sigma-Aldrich, Steinheim, Germany) was dissolved in methanol (99.8% pure, Sigma-Aldrich, Steinheim, Germany) to prepare a 1 mM stock solution, which was aliquoted, wrapped in aluminum foil (light-sensitive) and stored at 4°C. 5-Fluorouracil (>99% pure, Sigma-Aldrich, Steinheim, Germany) was dissolved in dimethyl sulfoxide (DMSO) to prepare a 28 mM stock solution and was stored at 4 °C. 

Water-insoluble tamoxifen and 5-fluorouracil (5-Fu) were used for nanoparticle loading purposes. The powder forms of 5-Fu and tamoxifen were loaded in excess. Before the experiment, excess 5-Fu and tamoxifen were allowed to sediment, and the final concentrations of treatment loaded nanopolymers were 3 μM for both large FA-DABA-SMA (350,000 g/mol) and small FA-DABA-SMA (20,000 g/mol) made in the standard culture medium. 

### 5.3. FA-DABA-SMA Alternating Copolymer

We previously reported on a novel polymer composed of a hydrophobic group (styrene) alternating with a hydrophilic group (maleic acid) along the polymeric chain (poly (styrene-*alt*-maleic anhydride) (SMA)). The polymer can self-assemble into nanostructures at physiological pH with a hydrophobic inner core that facilitates encapsulation of hydrophobic drugs [30,31]. The synthesis of this nanopolymer and characterization of this nanopolymer were reported by us [1,2,4]. For this study, two variations of this polymer were used to test the effects of size. The large polymer had a molecular weight of 350,000 g/mol (350 kDa) and a single chain hydrodynamic radius (Rh) of 6 nm and self-assembled into sheets (Rh of the self-assembled SMA structure of 850 nm in water). The small polymer had a molecular weight of 20,000 g/mol (20 kDa) with a single chain hydrodynamic radius (Rh) of 3 nm and self-assembled into cylinders (Rh of the self-assembled SMA structure of 120 nm in water). 

### 5.4. WST-1 Assay

The water-soluble tetrazolium salt-1 (WST-1) assay is a direct measure of metabolically active cells, based on the reduction of a tetrazolium compound to its soluble formazan (orange) derivative by metabolically active cells [32]. The absorbance of the reaction at 420 nm directly correlates to the number of living cells in culture. 

Cells were plated at a density of 10,000 cells/well in 96 well plates, incubated and allowed to adhere overnight. Adhered cells were treated with their respective concentrations of either curcumin loaded or empty FA-DABA-SMA or were left as the untreated controls. At the 24, 48 and 72 hour time-points following treatment (Days 1, 2 and 3, respectively), media was removed, and 100 μL of 10% WST-1 reagent (Roche Diagnostics, Laval-des-Rapides, QC, Canada) diluted in cell culture media were added to each well. The 96 well plate was then incubated at 37 °C for 2 hours before taking an absorbance reading using the SpectraMax250 machine and SoftMax software. Cell viability measured as a percentage of untreated control was illustrated as a bar graph using GraphPad Prism software (GraphPad Software, La Jolla, CA, USA). 

The following formula was used to determine cell viability as a percentage of control (Day 0) after 24, 48 and 72 hours of drug treatment (Days 1, 2 and 3, respectively):

$$\frac{\left[\left(absorbance\;in\;given\;drug\;concentration\;on\;day\;1,\;2\;or\;3\right)-\left(media\;absorbance\right)\right]\;\times 100}{\left(untreated\;absorbance\;on\;day\;0\right)-\left(media\;absorbance\right)}$$

### 5.5. Immunocytochemistry

DU-145, PANC-1 and MDA-MB-231 cancer cells were plated at a density of 100,000 cells/mL on glass coverslips in 24 well plates. Cells were treated for 48 hours with empty FA-SMA-DABA or were blocked with FR antibody. At the end of the time point, cells were washed and fixed with 4% PFA for 30 minutes followed by blocking for 1 hour in 10% FBS + 0.1% Triton X-100 + 1× PBS (note: 0.1% Triton x-100 was omitted from blocking buffer for membrane only stains to prevent intracellular non-specific binding). Following blocking, cells were washed with 1× PBS 3× for 10 minutes, followed by the addition of the primary antibody, which was diluted to 1:250 using a 1% FBS + 1×PBS + 0.1% Triton X-100 (note: 0.1% Triton X-100 was omitted from antibody buffer for membrane only stains to prevent intracellular non-specific binding) overnight at 4 °C. Primary antibodies used for these studies were FR (sc-515521), STAT-3 (sc-8019), p53 (Bethyl Laboratories Inc., Montgomery, TX 77356 USA, A300-249A-T), phospho-p53 (R&D Systems, Minneapolis, MN 55413, USA) and c-Myc (sc-40). Cells were washed 5× for 10 minutes with 1× PBS and incubated for 1 hour with AlexaFluor 594 secondary antibodies. Cells were then washed 5× for 10 minutes with 1× PBS (note: one wash included 0.1% Triton X-100 to permeabilize cells for DAPI). DAPI containing mounting media (VECTH1200, MJS BioLynx Inc., P.O. Box 1150, 300 Laurier Blvd., Brockville, ON K6V 5W1, Canada) was added to slides, and coverslips were inverted on to the mounting media droplet and sealed. 

### 5.6. Relative Fluorescence Density Quantification 

Relative fluorescence density was quantified using images captured at 400× to ensure a wide field of view was obtained. Two representative images were taken at 400×. Background mean, mean and pixel measurements were obtained from Corel Photo-Paint X8. Red (AlexaFluor 594, Abcam Inc., c/o 913860, PO Box 4090 Stn A, Toronto, ON M5W 0E9, Canada) or green (AlexaFluor 488, Abcam Inc., c/o 913860, PO Box 4090 Stn A, Toronto, ON M5W 0E9, Canada) color channel images were quantified. The background means represent an unstained section of the image, and the mean represents the total fluorescence of the image. These measurements were then used to quantify the relative fluorescence density using the equation below:

$$density=\;\left(bkgd\;mean-mean\right)\times pixels$$

The relative fluorescence density was then corrected for unspecific staining by subtracting the relative fluorescence density of the secondary antibody control images. These corrected values are represented in Figure 1, Figure 5, Figure 6, Figure 7 and Figure 8.

### 5.7. Immunofluorescence Live Cell Tracking 

Cells were plated at a density of 150,000 cell/well on glass coverslips in 24 well plates. Cells were treated for 12, 24 and 48 hours with 300 μL of curcumin loaded FA-DABA-SMA. Curcumin represents a hydrophobic drug model in addition to acting as a fluorescent probe. At the end of each time point, glass coverslips were removed from the 24 well plates. Live cells were treated with the Hoechst 33342 nuclear counterstain to visualize nuclei and curcumin loaded FA-DABA-SMA at the respective time points. DAPI containing mounting media (ab104139, Abcam Inc., c/o 913860, PO Box 4090 Stn A, Toronto, ON M5W 0E9, Canada) diluted with 1× PBS + 0.1% Triton X-100 was added to slides, and coverslips were inverted on to the mounting media droplet. Pictures were taken at the respective time points using the green (AlexaFluor 488) color channel.

### 5.8. Caspase Assay

Cells were plated at a density of 100,000 cells/well on glass coverslips in 24 well plates. Cells were treated for 24 and 48 hours with 300 μL of empty FA-DABA-SMA. At the end of each time point, caspase staining (CellEvent™ Caspase 3/7 Green Detection Reagent, Thermo Fisher Scientific, 168 Third Avenue, Waltham, MA 02451, United States) was diluted to the manufacturer’s recommended concentration of 5 μM in 5% FBS + 1× PBS from the stock solution of 2 mM. Stained cells were incubated for 30 minutes, then washed 2× in 1× PBS. The cells were then fixed with 4% paraformaldehyde solution diluted in 1× PBS for another 30 minutes. DAPI containing mounting media (Abcam ab104139) diluted with 1× PBS + 0.1% Triton X-100 was added to slides, and coverslips were inverted on to the mounting media droplet. Images were taken at the respective time points using the green (AlexaFluor 488) color channel. 

### 5.9. Flow Cytometry

DU-145, PANC-1 and MDA-MB-231 cells were harvested and counted for a final concentration of 1.0 × 10^6^ cells/mL. All subsequent steps were done on ice. Cells were washed 2× in 2% FBS + 1× PBS before primary antibody addition. The cells were treated with 100 μL of FR (sc-515521, Santa Cruz Biotechnology, Inc., 10410 Finnell Street, Dallas, Texas 75220, U.S.A.) primary monoclonal mouse antibody at a final concentration of 10 μg/mL and incubated for 60 minutes. Secondary control cells were treated with 100 μL of 2% FBS + 1× PBS and incubated for 60 minutes. The cells were then washed 2× with 2% FBS + 1× PBS followed by incubation for 60 minutes with 100 μL of secondary anti-mouse antibody AlexaFluor 488 at a final concentration of 10 μg/mL. The cells were then washed 2× with 2% FBS + 1× PBS and fixed in 1 mL of 4% paraformaldehyde solution before flow cytometry analysis. 

### 5.10. Annexin V Assay

PANC-1 and MDA-MB-231 were plated at a density of 1 × 10^6^ cells/mL in a T25 tissue culture flask. Cells were treated with empty FA-DABA-SMA for 48 hours or left untreated. The cells were then trypsinized, and ~250,000 cells/mL were analyzed for apoptotic, necrotic and viable cell populations using the Annexin V-FITC Apoptosis Kit (K101-25, BioVision, Inc., 155 S Milpitas Blvd. Milpitas, CA 95035, USA) following the manufacturer’s manual. Briefly, the collected cells were resuspended in 500 μL of 1× binding buffer followed by 5 μL of each Annexin V-FITC and propidium iodide. The cells were incubated for 5 minutes at room temperature in the dark, followed by quantification by a flow cytometer. 

### 5.11. Folic Receptor Blocking 

PANC-1 and MDA-MB-231 cancer cells were plated at a density of 100,000 cells/mL on glass coverslips in 24 well plates for immunocytochemistry experiments or at a density of 10,000 cells/well for WST-1 experiments. For both experiments, cells were treated for 2 hours with the FR (sc-515521) antibody, followed by treatment with large empty nanopolymer. WST-1 experiments were performed according to the procedure outlined in Section 5.4*,* and immunocytochemistry experiments were performed after 48 hours of treatment following the procedure outlined in Section 5.5. 

### 5.12. Statistical Analysis

Data are presented as the means ± the standard error of the mean (SEM) from two repeats of each experiment, each performed in triplicate. All statistical analyses were performed with GraphPad Prism software. All results were compared with one-way analysis of variance (ANOVA) and Fisher’s LSD test, with the following asterisks denoting statistical significance: * *p* ≤ 0.05, ** *p* ≤ 0.001 and *** *p* ≤ 0.0001.
